# Machine Learning Revealed New Correlates of Chronic Pelvic Pain in Women

**DOI:** 10.3389/fdgth.2020.600604

**Published:** 2020-12-18

**Authors:** Mohamed Elgendi, Catherine Allaire, Christina Williams, Mohamed A. Bedaiwy, Paul J. Yong

**Affiliations:** ^1^School of Electrical and Computer Engineering, University of British Columbia, Vancouver, BC, Canada; ^2^Faculty of Medicine, University of British Columbia, Vancouver, BC, Canada; ^3^British Columbia (BC) Children's & Women's Hospital, Vancouver, BC, Canada

**Keywords:** obstetrics, gynecology, chronic pelvic pain in women, endometriosis, quality of life, infertility, data science, artificial intelligence

## Abstract

Chronic pelvic pain affects one in seven women worldwide, and there is an urgent need to reduce its associated significant costs and to improve women's health. There are many correlated factors associated with chronic pelvic pain (CPP), and analyzing them simultaneously can be complex and involves many challenges. A newly developed interaction ensemble, referred to as INTENSE, was implemented to investigate this research gap. When applied, INTENSE aggregates three machine learning (ML) methods, which are unsupervised, as follows: interaction principal component analysis (IPCA), hierarchical cluster analysis (HCA), and centroid-based clustering (CBC). For our proposed research, we used INTENSE to uncover novel knowledge, which revealed new interactions in a sample of 656 patients among 25 factors: age, parity, ethnicity, body mass index, endometriosis, irritable bowel syndrome, painful bladder syndrome, pelvic floor tenderness, abdominal wall pain, depression score, anxiety score, Pain Catastrophizing Scale, family history of chronic pain, new or re-referral, age when first experienced pain, pain duration, surgery helpful for pain, infertility, smoking, alcohol use, trauma, dysmenorrhea, deep dyspareunia, CPP, and the Endometriosis Health Profile for functional quality of life. INTENSE indicates that CPP and the Endometriosis Health Profile are correlated with depression score, anxiety score, and the Pain Catastrophizing Scale. Other insights derived from these ML methods include the finding that higher body mass index was clustered with smoking and a history of life trauma. As well, sexual pain (deep dyspareunia) was found to be associated with musculoskeletal pain contributors (abdominal wall pain and pelvic floor tenderness). Therefore, INTENSE provided expert-like reasoning without training any model or prior knowledge of CPP. ML has the potential to identify novel relationships in the etiology of CPP, and thus can drive innovative future research.

## 1. Introduction

Chronic pelvic pain affects nearly 15% of women, with major impact on quality of life and health care costs (1, 2). The etiology of chronic pelvic pain (CPP) is very complex, involving many interrelated and correlated factors over the course of one's life, including the presence or absence of endometriosis. Recently, Yosef et al. (3) performed an exploratory analysis of multifactorial variables independently associated with the severity of CPP in women. Among the findings, they found that abdominal wall pain (i.e., pain related to the abdominal wall musculature), tenderness of the pelvic floor musculature, and Pain Catastrophizing Scale were independently associated with the severity of CPP with significance of *p* ≤ 0.05, but surprisingly, no association with endometriosis. However, the authors used multiple linear regression and thus did not investigate the simultaneous dynamics between factors. In complex clinical conditions, such as CPP, straightforward regression analyses may provide an incomplete view of the impact of each factor in relation to other factors.

It is our understanding that, as it currently stands, minimal effort has been made to examine different factors simultaneously using artificial intelligence (AI), with a focus on the network dynamics between potential factors, in this area of medicine. Thus, in this study, we utilize AI-informed machine learning (ML) methods to uncover the hidden interactions among all factors and explore the importance of each factor for CPP in women.

## 2. Materials and Methods

### 2.1. Pelvic Pain and Endometriosis Dataset

This study is a re-analysis of cross-sectional data from Yosef et al. (3) (*N* = 656 subjects), which are taken from a prospective database from a tertiary referral center for pelvic pain and endometriosis using the REDCap system (4, 5). Participants from December 2013 to September 2015 were included, who completed an online questionnaire and underwent a complete history/examination. Exclusion criteria included age > 50 or menopausal (6). The sample characteristics have been published previously (3), with a mean (± 1 standard deviation) age of 34.5 (±7.6) years and body mass index (BMI) of 25.3 (±5.7) kg/m^2^, with 49% of the sample nulligravid and 74% of the sample Caucasian, who had underlying diagnoses of endometriosis (57%), irritable bowel syndrome (53%), painful bladder syndrome (43%), and abdominal wall trigger points (27%). We chose 25 factors of clinical importance in this cohort based on the initial analysis of Yosef et al. (3), as shown in Table 1.

**Table 1:** The 25 factors of clinical importance to chronic pelvic pain used in this study.

**Variable name**	**Description**
Age	Years
Parity	Nulliparous (no childbirth) vs. Parous (at least one childbirth)
Ethnicity	Other vs. Caucasian
Body mass index (BMI)	*kg*/*m*^2^
Endometriosis (3)	Absent/not suspected (prior negative laparoscopy or not clinically suspected) vs. clinically suspected (based on history and/or tenderness on examination (6)) vs. confirmed present (prior surgical diagnosis or current nodule or endometrioma on exam/imaging)
Irritable bowel syndrome (IBS) (7)	Present vs. Absent
Painful bladder syndrome (PBS) (8)	Present vs. Absent
Pelvic floor tenderness	Tenderness of the levator ani pelvic floor musculature on examination, as a sign of myofascial pelvic pain syndrome: Present vs. Absent
Abdominal wall pain	Abdominal wall pain diagnosed by the Carnett test (1), with abdominal tenderness not changing or worsening with tensing of the abdominal wall musculature, often secondary to myofascial trigger points: Present vs. Absent
Depression	Patient Health Questionnaire-9 questionnaire (9)
Anxiety	Generalized Anxiety Disorder-7 questionnaire (10)
Pain catastrophizing	Pain Catastrophizing Scale (11) (measurement of magnification or rumination on symptoms, as well as feelings of helplessness)
Family history of chronic pain	Yes vs. No vs. Do not know
Referral type	New or re-referral
Age when first experienced pain	Years
Pain duration	Years
Patient report that prior surgery was helpful for pain	Yes vs. No vs. No prior surgery
Infertility	Yes vs. No vs. Never tried for pregnancy
Smoking	Yes vs. No
Alcohol use	Drinks/week
Trauma	Based on 7 questions about childhood or adult sexual, physical, or emotional abuse3 (scored from 0–7)
Dysmenorrhea	Menstrual cramps (rated 0–10)
Deep dyspareunia	Pain with deep penetration during sexual activity (rated 0–10)
Chronic pelvic pain	Chronic pain in the pelvic (rated 0–10)
Endometriosis Health Profile-30 (12)	indicating worse quality-of-life)

### 2.2. Pre-processing Step

To standardize the values of each factor, we applied the Z-score normalization. It is implemented by subtracting the mean from each factor, then divide the result by the standard deviation of each factor as follows: $F=\left(X-\bar{X}\right)/\left(\sigma \right),$

where *F* is the normalized factor vector, *X* is the raw factor vector, ($\bar{X}=\frac{1}{N}\overset{N}{\underset{n=1}{\sum }}X_{n}$) is the mean of the factor vector, ($\sigma =\frac{1}{N-1}\overset{N}{\underset{n=1}{\sum }}{\left(X_{n}-\bar{X}\right)}^{2}$) is the standard deviation of the factor vector, *N* is the number of subjects, which equals 656 in this work.

### 2.3. INTENSE Algorithm

INTENSE, a newly developed interaction ensemble method that utilizes various clustering models (13) was used. Multiple models for clustering are used in existing literature; however, each has its own set of rules for defining factors with “mathematical similarity.” When implementing the INTENSE method, results are aggregated from three different interaction methods, with a different mathematical view for each:

1. Interaction based on principal component analysis examines correlations between all factors based on eigen values.
2. Interaction based on connectivity clustering examines distance-wise similarity between each two factors.
3. Interaction based on centroid clustering examines distance-wise similarity between all factors and the proposed number of centroids.
4. INTENSE aggregates findings from the above interactions for a consensus regarding how all the factors in the dataset interact with each other.

Since each method for interactions has limitations, such as the initial value used, fixed thresholds, and so on, INTENSE was created. When combining results from various interaction models that utilized different geometrical concepts, the output will be an aggregate of agreed upon results, thus creating a more robust conclusion.

#### 2.3.1. Interaction Principal Component Analysis

A correlation-based machine learning method was used in this study, referred to as the IPCA, proposed in a previous study (13). As an unsupervised ML technique, within set of observations of attributes that are potentially correlated, it identifies linearly uncorrelated attributes (in this instance, factors). A decorrelation process is first used that does not need any initial conditions for the processed attributes. Next, the Pearson's correlation is applied. In the absence of any training of labeling, IPCA can automatically reveal hidden interactions between factors, and provide a true level of learning where new behaviors among the factors examined are uncovered. Algorithm 1 shows the pseudocode of IPCA.

**Algorithm 1:** Interaction Principal Component Analysis (IPCA).

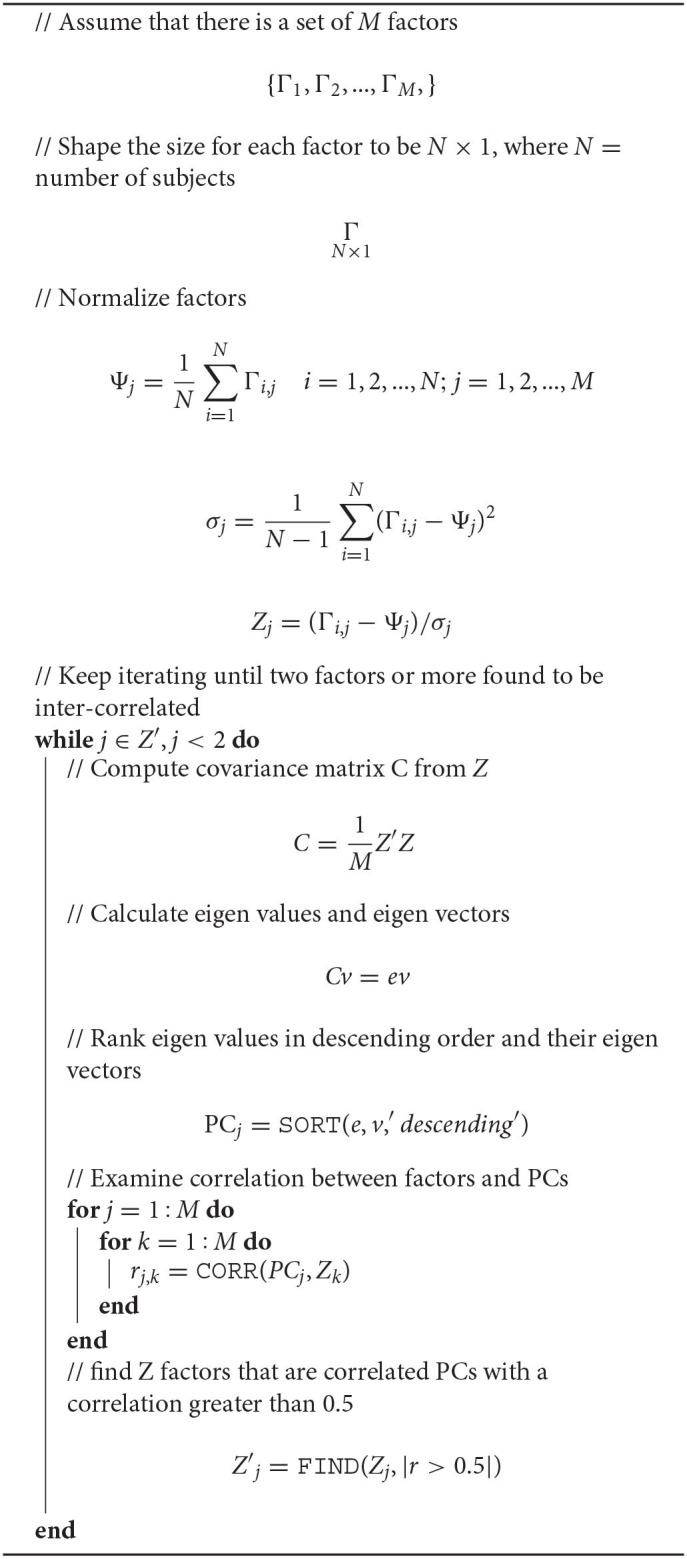

#### 2.3.2. Hierarchical Cluster Analysis

An unsupervised ML approach, termed the hierarchical cluster analysis (HCA), connects “factors” and based on their distance, groups are formed. Among biosignals, HCA can visualize and quantify dissimilarities. To provide a hierarchical cluster, the Euclidean distance *d* = ||*a*−*b*||_2_ was implemented, also referred to as the dendrogram. “Average” is utilized here as the linkage criterion to determine the distance between all factors as a function of the pairwise distances between observations,which is defined as $d\left(W,v\right)=\sum \frac{d\left(w\left[i\right],v\left[j\right]\right)}{\left|w|\times |v\right|}$, for all data points *i* and *j*, where |*w*| and |*v*| are the cardinalities of clusters |*w*| and |*v*|, respectively.

#### 2.3.3. Centroid-Based Clustering

A well-known and relatively simple centroid-based algorithm for clustering, known as the *K*-means clustering (centroid-based clustering, CBC) is used here. The number of factors *F* is divided into *K* disjoint clusters. The statistical means of a group of factors form clusters. In other words, the factors with minimum distance between them and their statistical mean formulate an independent cluster. To find the minimum distance between a group of factors and their corresponding statistical mean, the within-cluster sum-of-squares, also known as inertia, is commonly used. Inertia is defined as: $||f_{i}-\mu_{j}|{|}^{2}$, where *f*_*i*_ is all values in factor *i*, and μ_*j*_ refers to the mean of all factors in cluster *j*. It is highly recommended to apply PCA before CBC clustering to reduce dimensionality and visualize the results in two dimensions.

#### 2.3.4. Ensemble Method

We used the “majority voting” rule to combine conceptually different interaction recommendations by different methods. In other words, in majority voting, the consistent interactions suggested by different clustering methods are the ideal and more meaningful interactions. For example, if the recommendations are

1. IPCA ↦ C1 (*f*_1_, *f*_3_), C2 (*f*_5_, *f*_7_), C3 (*f*_8_, *f*_9_, *f*_10_)
2. HCA ↦ C1 (*f*_1_, *f*_3_, *f*_5_), C2 (*f*_8_, *f*_9_)
3. CBC ↦ C1 (*f*_1_, *f*_3_, *f*_6_), C2 (*f*_6_, *f*_7_)

then the ensemble decision is that *f*_1_ and *f*_3_ interact strongly and more strongly correlated among all other factors.

### 2.4. Software

We used Python 3.6.5 software and Matlab 2018b software to analyze the data.

## 3. Results

**Figure 1:**
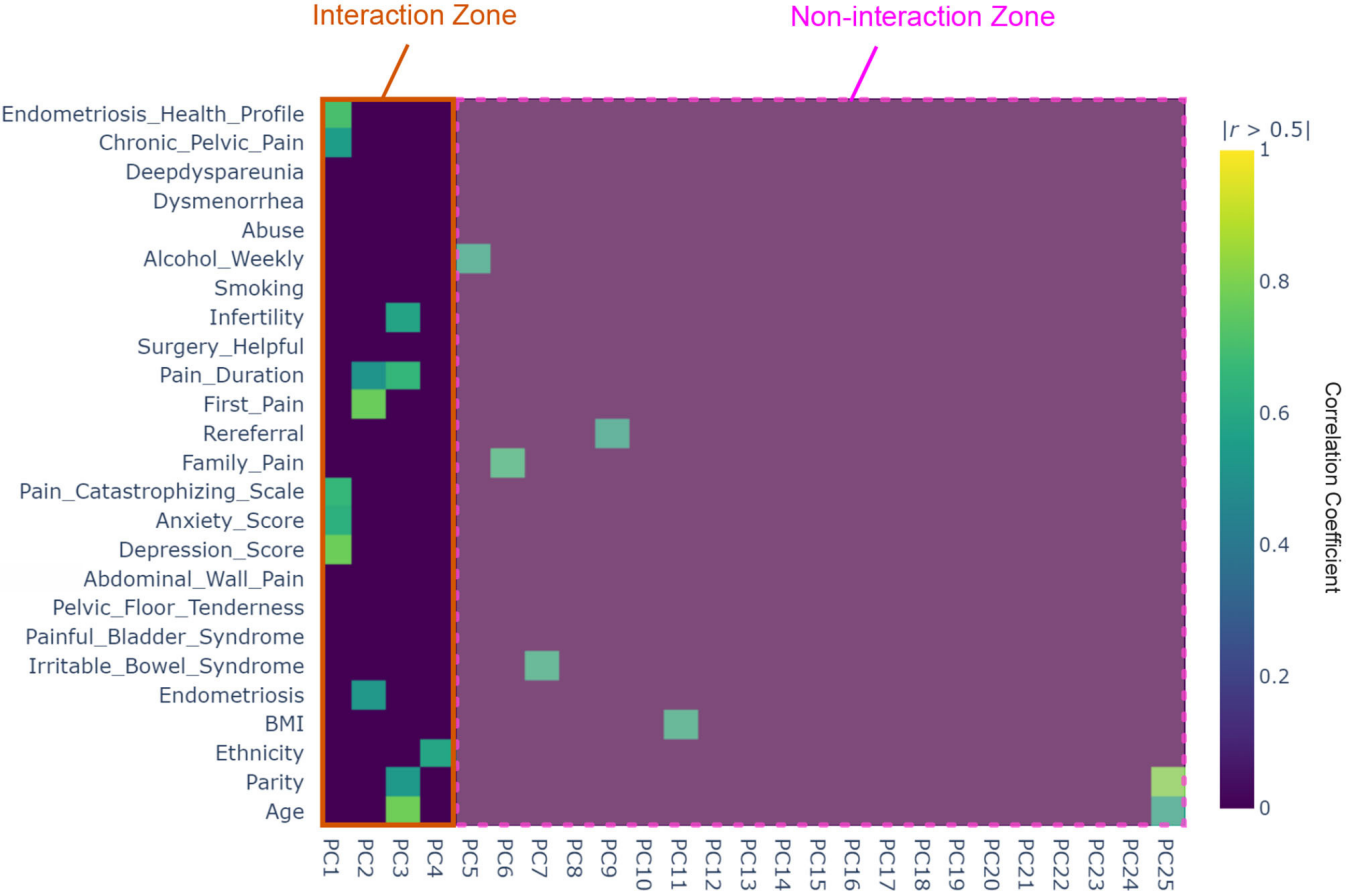
Heat map of the interaction principle component analysis (IPCA) based on the determined 25 factors from the Pelvic Pain and Endometriosis database. The interaction zone refers to the number of principle components (PCs) where two or more factors are correlated, while the non-interaction zone refers to the number of PCs that have only single factor that is not correlated with any other factors.

The significance of the 25 principal components extracted from the database are shown in Figure 1. Most of the variance is explained by PC1 (40%), which reflects the relevance and importance of factors correlated with PC1; 23% of the variance is explained by PC2, and lastly ~17% of variance is explained by PC3. It can be seen that PC1 is the most important, followed by PC2. PC25 shows to be the least important.

A correlation matrix heat map, shown in Figure 1, demonstrates the interaction between factors and all PCs. Diagonal entries are equal to one. There are four 25 × 25 blocks. The correlation matrix for PCs in the top right block contains zeros, confirming that the principle components (PCs) are mutually orthogonal, and hence are not correlated. Correlation between all factors is shown in the bottom-left block, and thus, there no correlation was reported.

**Figure 2:**
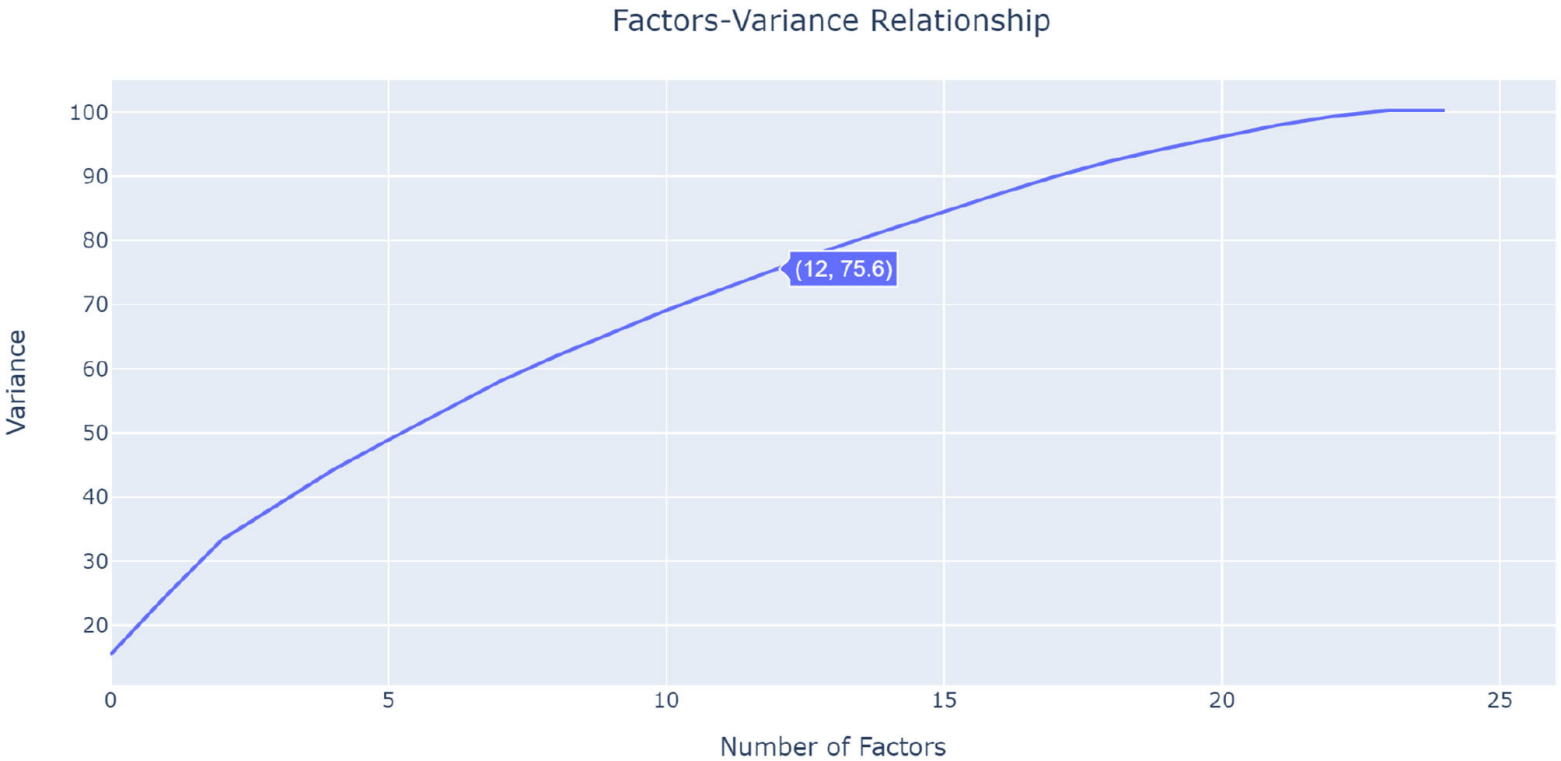
Number of dimensions needed to account for 75% of the total variance.

As seen in Figure 1, the 25 × 25 heatmap contains interesting results about the factors interaction. IPCA involves two steps: First, it identifies the most strongly interacting factors, following which IPCA is run again on theses selected factors. In the first step, IPCA identified 12 factors that are interacting with each other: age, parity, endometriosis, depression score, anxiety score, Pain Catastrophizing Scale, first pain, pain duration, infertility, CPP, and Endometriosis Health Profile. These 12 factors are located within the first four PCs (PC1–4), which are located in the interaction zone in Figure 1. The IPCA algorithm found that there is no interaction between PCs and factors after PC4; therefore, it used only the factors associated with the first four PCs. This was confirmed by running a cumulative sum for all PCs. Figure 2 visualizes the cumulative sum of PCs and shows that 12 dimensions (i.e., PCs) are needed to account for 75% of the total variance, which is above the 70% cut-off point (14) for determining the optimal number of PCs. Note that the non-interaction zone shown in Figure 1 contains only individual factors that are not interacting with other factors.

**Figure 3:**
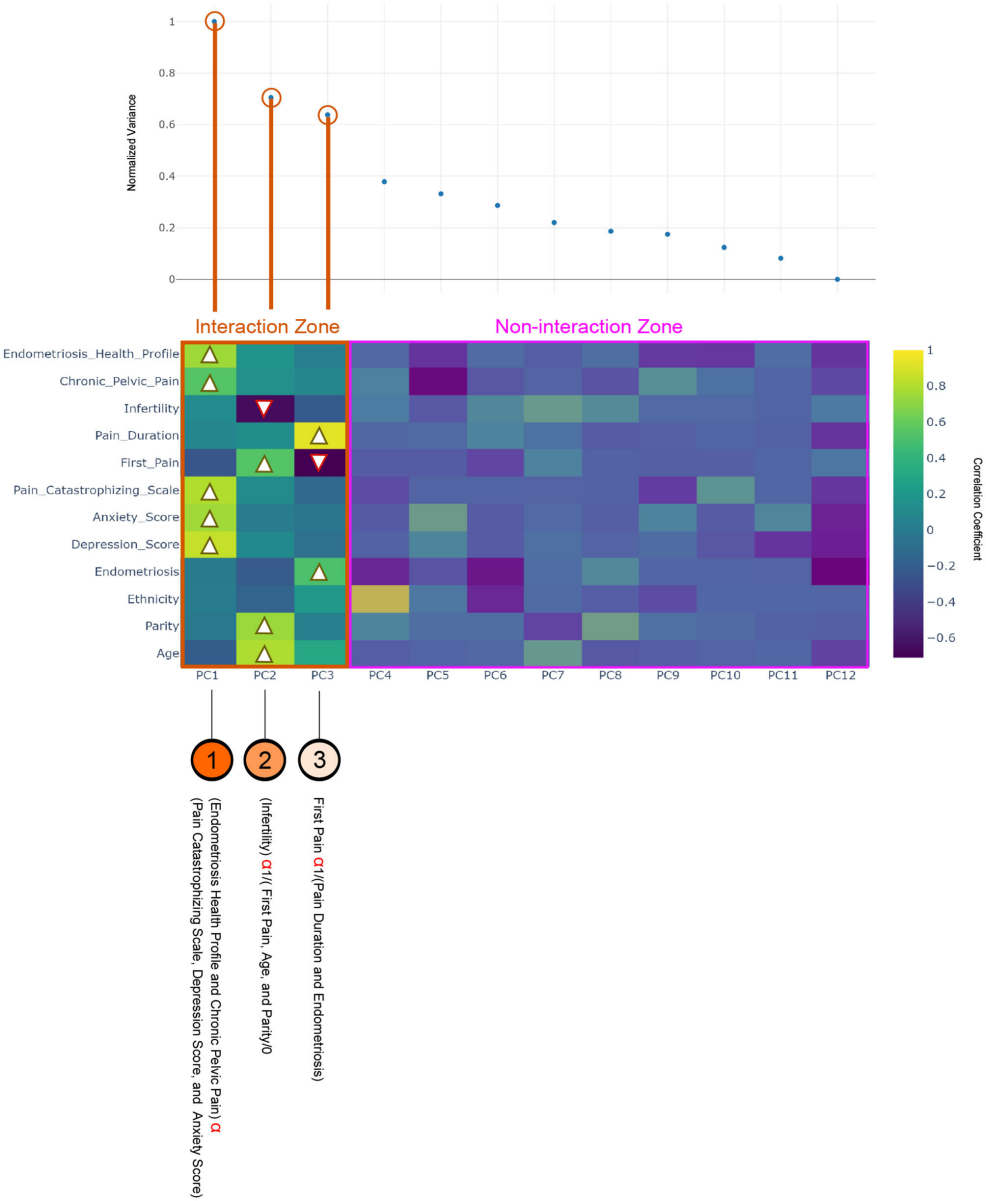
Heat map of the interaction principle component analysis (IPCA) between 12 factors using the Pelvic Pain and Endometriosis database, and the black arrow indicates the direction of the correlation. The interaction zone refers to the number of principle components (PCs) where two or more factors are correlated, while the non-interaction zone refers to the number of PCs that have only single factor that is not correlated with any other factors.

The last step of IPCA shows the interactions between the previously determined 12 factors. As shown in Figure 3, the first column shows significant correlation-based interactions between PC1 and CPP, Endometriosis Health Profile, Pain Catastrophizing Scale, anxiety score, and depression score. Chronic pelvic pain, Endometriosis Health Profile, Pain Catastrophizing Scale, anxiety score, and depression score factors move in the same direction. Clinically, this suggests that higher CPP severity, worse quality-of-life, and more anxiety, depression, and pain catastrophizing, all correlate with each other.

In the second column age, and age at first pain, and parity are moving together, which is another kind of AI produced by IPCA, and indicates that younger age, earlier age at first pain, and nulliparity are all correlated. In contrast, these variables are strongly inversely correlated with infertility, which suggests that having never tried for pregnancy is associated with younger age, earlier age at first pain, and nulliparity. The third column shows that endometriosis and pain duration move in the same direction, and both are inversely correlated with age at first pain. This is another level of AI produced by IPCA, and demonstrates that those with younger age at first pain have a longer pain duration and also are more likely have a confirmed diagnosis of endometriosis. PC4–12 show no interactions between factors.

**Figure 4:**
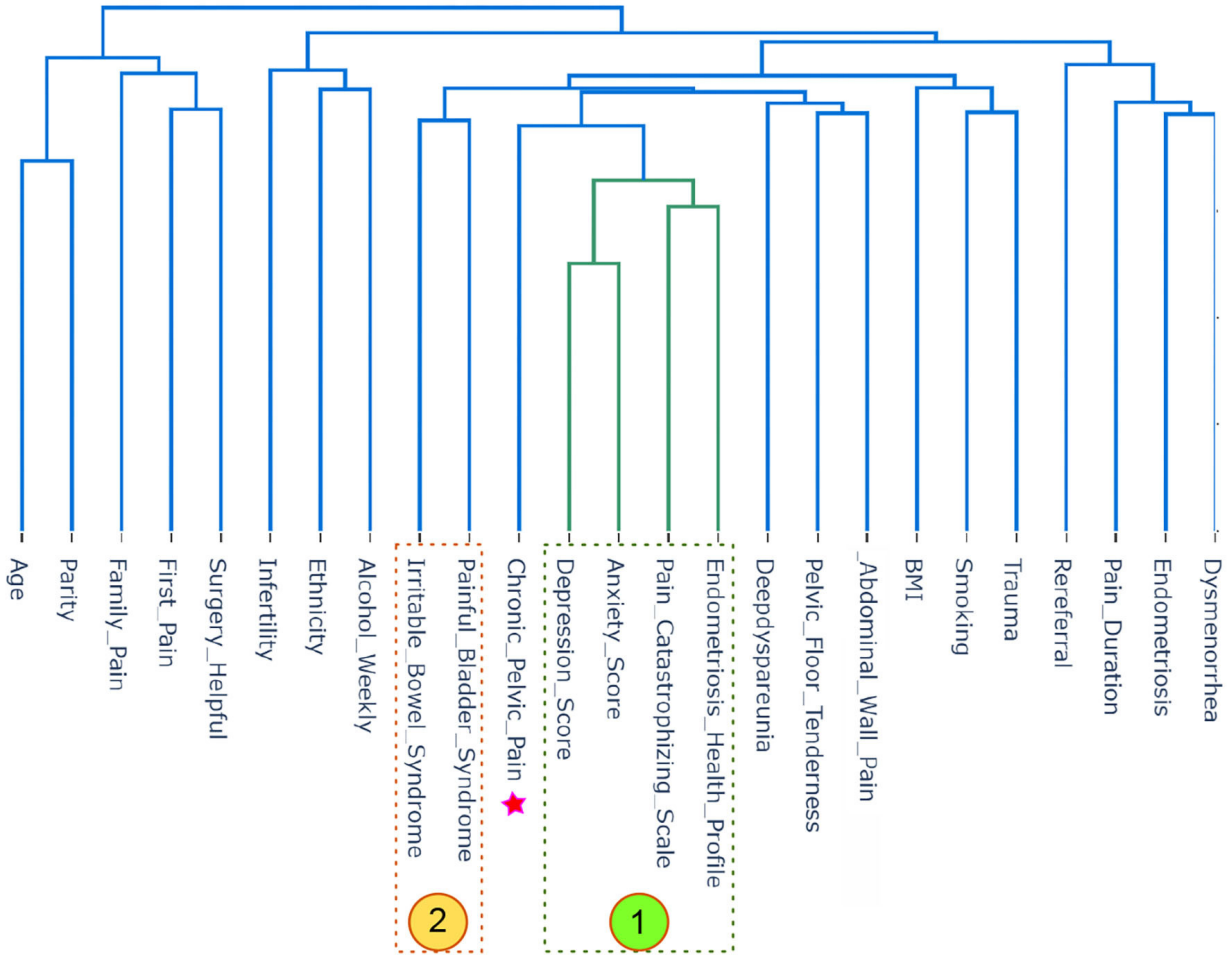
Hierarchical cluster analysis (HCA) analysis of all 25 factors in the Pelvic Pain and Endometriosis database. The star represents the factor of interest, which is chronic pelvic pain (CPP). Four factors in zone #1 are directly associated with CPP, while two factors in zone #2 are indirectly associated with CPP.

The interaction based on hierarchical clustering of factors is shown in the dendrogram. A hierarchy is built that progressively merges the independent factors to generate clusters. The 25 factors were used, and the process works based on determining how close each set of two factors are. The factors were clustered according to their similarity, as shown in Figure 4. By visually inspecting Figure 4 shows that the changes in the CPP and Endometriosis Health Profile are similar and are both clustered with anxiety score, depression score, and Pain Catastrophizing Scale. This is a kind of AI recommendation produced by HCA, and it suggests that the anxiety score, depression score, and Pain Catastrophizing Scale are good correlates for CPP and Endometriosis Health Profile. This finding is in agreement with the IPCA finding, as shown in Figure 3.

As can be seen in Figure 4, the most interesting recommendation produced using HCA is clustering alcohol, ethnicity and infertility as one group, such that infertility was correlated with non-Caucasian ethnicity and less alcohol use. Note that HCA was able to detect a non-linear correlation compared to the traditional linear bivariate correlation that was not able to detect a correlation between ethnicity and alcohol (i.e., *r* = 0.158), ethnicity and infertility (i.e., *r* = 0.068), and alcohol and infertility (i.e., *r* = 0.065).

Interestingly, as shown in Figure 4, HCA grouped BMI with trauma and smoking, suggesting a correlation between the three (patients who are smoking and have been traumatized have higher BMI in this database). In fact, HCA showed that trauma and smoking are together directly associated with BMI. Note that HCA was able to detect a non-linear correlation compared to the traditional linear bivariate correlation that was not able to detect a correlation between trauma and BMI (i.e., *r* = 0.0176), trauma and smoking (i.e., *r* = 0.217), and BMI and smoking (i.e., *r* = 0.108).

**Figure 5:**
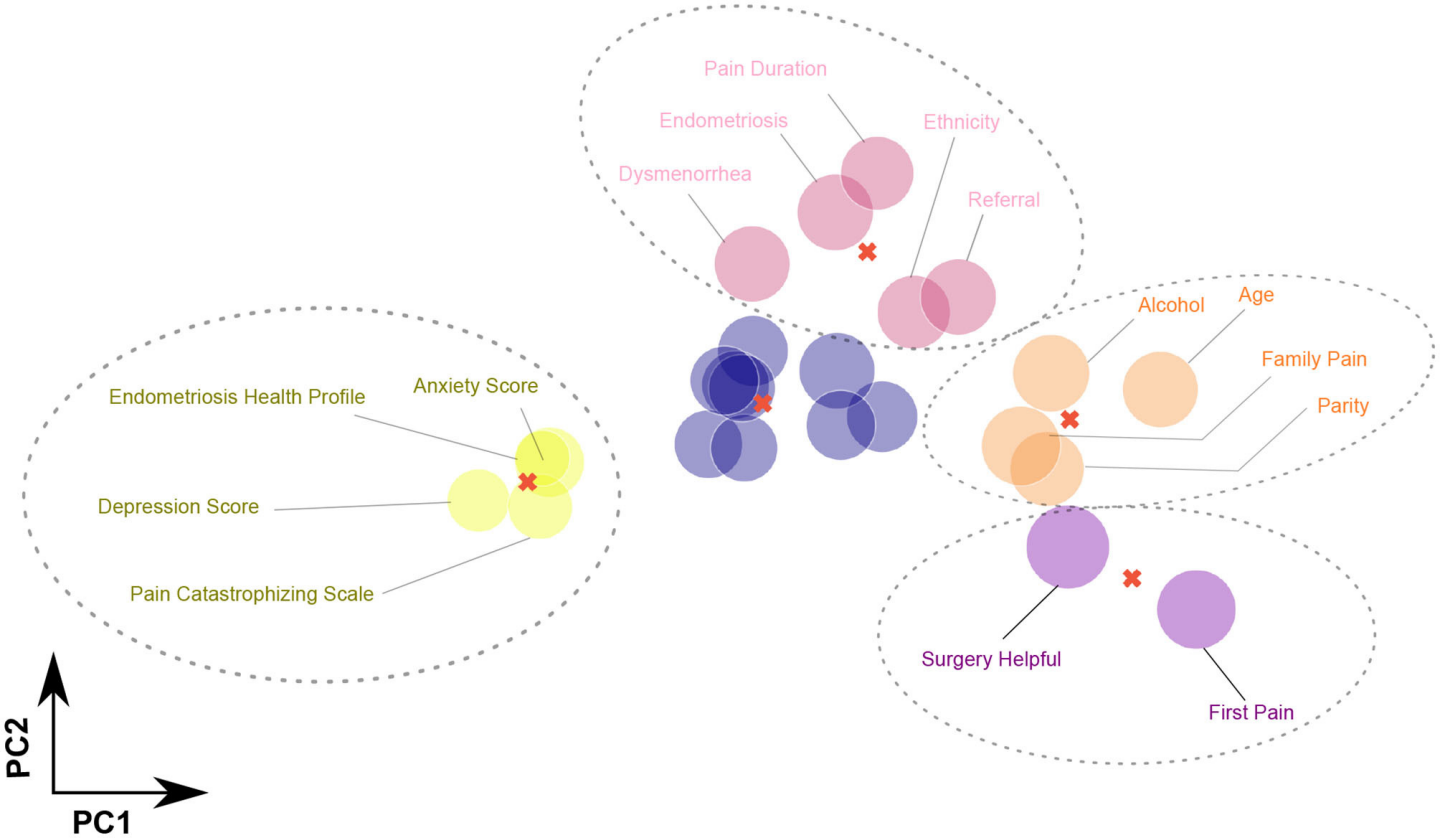
Centroid-based clustering (CBC) analysis of all 25 factors in the Pelvic Pain and Endometriosis database using five clusters. Four factors (Pain Catastrophizing Scale, anxiety score, depression score, and Endometriosis Health Profile) are formulated in one cluster with respect to chronic pelvic pain (CPP). Also, four factors (age, family history of chronic pain, parity, and alcohol use per week) are formulated in one cluster with respect to CPP. Nine factors [body mass index (BMI), infertility, trauma, deep dyspareunia, smoking, irritable bowel syndrome, painful bladder syndrome, pelvic floor tenderness, and abdominal wall pain] are formulated in one cluster with respect to CPP. Interestingly, age at first pain and history of surgery are correlated with each other with respect to CPP.

The third geometrical interaction for our factors is CBC, which represents an alternative clustering method. Initially, CBC requires the desired number of clusters to process the data. We tested the inertia and found that the ideal number of clusters that reduces the distance between factors and their centroids is five. Then CBC was set up with five clusters, with respect to CPP, CBC clustered anxiety score, Pain Catastrophizing Scale, depression score, and Endometriosis Health Profile as one cluster, the first cluster on the left side of Figure 5. This finding is in agreement with the IPCA and HCA findings, as shown in Figures 3, 4, respectively.

Also, CBC clustered four factors (age, family history of chronic pain, parity, and alcohol use per week) as one cluster with respect to CPP. Note that IPCA and HCA confirmed the correlation between age and parity (older age and higher parity/more deliveries), and CBC is in agreement with this finding. In addition, CBC clustered nine factors (BMI, infertility, trauma, deep dyspareunia, smoking, irritable bowel syndrome, painful bladder syndrome, pelvic floor tenderness, and abdominal wall pain) as one cluster with respect CPP. Note that CBC clustered BMI with trauma and smoking, confirming the effects of BMI on smoking and trauma, which is in agreement with HCA finding. Moreover, CBC clustered age when first experienced pain with surgery being helpful (younger age when first experienced pain associated with surgery having been helpful).

It is worth mentioning that IPCA found CPP to be interacting with Pain Catastrophizing Scale, anxiety score, depression score, and Endometriosis Health Profile. HCA showed an indirect (placing both in the same group, but not close to each other) association between irritable bowel syndrome, painful bladder syndrome, and CPP. Both irritable bowel syndrome and painful bladder syndrome were placed on the left side of CPP in a different group, as shown in Figure 4. Interestingly, HCA showed an association between abdominal wall pain, pelvic floor tenderness, and deep dyspareunia. This points toward the importance of musculoskeletal contributors (abdominal wall trigger points and myofascial pelvic pain syndrome of the pelvic floor) to sexual pain.

## 4. Discussion

In this study, we utilized ML approaches to characterize factors that are correlated with CPP. To achieve this, we compared our results with those of a previously published study on the same dataset. The previous study (3) from our group on independent associations with factors suggested that seven factors were correlated with CPP: BMI, abdominal wall pain, pelvic floor tenderness, Pain Catastrophizing Scale, painful bladder syndrome, smoking, and history of adult trauma. However, these results showed the independent importance of each factor for chronic pelvic pain assessment. Our simultaneous analysis using INTENSE found that CPP and Endometriosis Health Profile are correlated with depression score, anxiety score, and Pain Catastrophizing Scale.

It was notable that endometriosis was not associated with chronic pelvic pain, as reported in our previous study (3) using regression analyses. However, in this current study, IPCA found an interesting collective relationship, where PC3 shows that those with younger age at first pain are more likely to have had surgery for endometriosis, which was reported as helpful. This was an interesting kind of AI observation, produced using IPCA. Clinically this makes sense, as patients with earlier onset pain and longer pain duration are more likely to undergo surgery to confirm the diagnosis and treat the endometriosis.

Our ML approach was also able to identify other unique relationships that were not apparent with routine regression analyses on the same dataset (3). For example, higher BMI was associated with a history of life trauma (15) and smoking (Figure 3). While the factors underlying this relationship are complex, one hypothesis is that life trauma could predispose to smoking as well as lifestyle habits that give risk to obesity. This hypothesis warrants further study.

Another interesting finding was that HCA clustered abdominal wall pain and myofascial pelvic pain of the pelvic floor musculature with deep dyspareunia (sexual pain) (Figure 4). This points to the importance of musculoskeletal factors in the etiology of sexual pain specifically, among women with pelvic pain. The same relationship with musculoskeletal factors was not seen for dysmenorrhea (menstrual cramps), indicating that dysmenorrhea may have a different pathophysiology compared to sexual pain. These unique aspects of the etiology of different types of pelvic pain, discovered using ML, also warrant future study.

A limitation of the study is the inherent heterogeneity of chronic pelvic pain, where multiple underlying diagnoses can be present. While the sample size (>500) helps to capture this heterogeneity in part, additional multi-center research is needed with even larger sample sizes given the complex multifactorial nature of chronic pelvic pain.

## 5. Conclusion

In this study, we have described our evaluation of the impact of chronic pelvic pain on various factors using machine learning approaches. INTENSE can to detect complex relationships between different factors for chronic pelvic pain, without the need for any previous training or knowledge, and is a completely unsupervised interaction method. The results of the ML methods showed agreement on the significant correlation between chronic pelvic pain and Endometriosis Health Profile-30, depression score, anxiety score, and Pain Catastrophizing Scale. Other unique relationships were also identified with ML, which provide data to drive future research.

## Data Availability Statement

The data analyzed in this study is subject to the following licenses/restrictions: available upon request. Requests to access these datasets should be directed to PY, paul.yong@vch.ca.

## Ethics Statement

The studies involving human participants were reviewed and approved by University of British Columbia. The patients/participants provided their written informed consent to participate in this study.

## Author Contributions

ME, CA, CW, MB, and PY conceived the study and drafted the manuscript. ME developed the INTENSE algorithm. All authors approved the final manuscript.

## Conflict of Interest

The authors declare that the research was conducted in the absence of any commercial or financial relationships that could be construed as a potential conflict of interest.
